# A Prediction Model to Identify Clinically Relevant Medication Discrepancies at the Emergency Department (MED-REC Predictor): Development and Validation Study

**DOI:** 10.2196/55185

**Published:** 2024-11-27

**Authors:** Greet Van De Sijpe, Matthias Gijsen, Lorenz Van der Linden, Stephanie Strouven, Eline Simons, Emily Martens, Nele Persan, Veerle Grootaert, Veerle Foulon, Minne Casteels, Sandra Verelst, Peter Vanbrabant, Sabrina De Winter, Isabel Spriet

**Affiliations:** 1 Pharmacy Department University Hospitals Leuven Leuven Belgium; 2 Clinical Pharmacology and Pharmacotherapy, Department of Pharmaceutical and Pharmacological Sciences KU Leuven Leuven Belgium; 3 Department of Gynaecology and Obstetrics University Hospitals Leuven Leuven Belgium; 4 Pharmacy Department General Hospital Sint-Jan Brugge-Oostende AV Brugge Belgium; 5 Department of Emergency Medicine University Hospitals Leuven Leuven Belgium

**Keywords:** medication reconciliation, medication discrepancy, emergency department, prediction model, risk stratification, MED-REC predictor, MED-REC, predictor, patient, medication, hospital, software-implemented prediction model, software, geographic validation, geographic

## Abstract

**Background:**

Many patients do not receive a comprehensive medication reconciliation, mostly owing to limited resources. We hence need an approach to identify those patients at the emergency department (ED) who are at increased risk for clinically relevant discrepancies.

**Objective:**

The aim of our study was to develop and externally validate a prediction model to identify patients at risk for at least 1 clinically relevant medication discrepancy upon ED presentation.

**Methods:**

A prospective, multicenter, observational study was conducted at the University Hospitals Leuven and General Hospital Sint-Jan Brugge-Oostende AV, Belgium. Medication histories were obtained from patients admitted to the ED between November 2017 and May 2022, and clinically relevant medication discrepancies were identified. Three distinct datasets were created for model development, temporal external validation, and geographic external validation. Multivariable logistic regression with backward stepwise selection was used to select the final model. The presence of at least 1 clinically relevant discrepancy was the dependent variable. The model was evaluated by measuring calibration, discrimination, classification, and net benefit.

**Results:**

We included 824, 350, and 119 patients in the development, temporal validation, and geographic validation dataset, respectively. The final model contained 8 predictors, for example, age, residence before admission, number of drugs, and number of drugs of certain drug classes based on Anatomical Therapeutic Chemical coding. Temporal validation showed excellent calibration with a slope of 1.09 and an intercept of 0.18. Discrimination was moderate with a *c*-index (concordance index) of 0.67 (95% CI 0.61-0.73). In the geographic validation dataset, the calibration slope and intercept were 1.35 and 0.83, respectively, and the *c*-index was 0.68 (95% CI 0.58-0.78). The model showed net benefit over a range of clinically reasonable threshold probabilities and outperformed other selection criteria.

**Conclusions:**

Our software-implemented prediction model shows moderate performance, outperforming random or typical selection criteria for medication reconciliation. Depending on available resources, the probability threshold can be customized to increase either the specificity or the sensitivity of the model.

## Introduction

Medication discrepancies occur frequently upon admission to the emergency department (ED) and are a major source of avoidable harm [1-5]. Taking an accurate medication history early on during the patient trajectory is the typical first step needed to tackle medication safety in a multifaceted approach. Medication reconciliation is necessary to identify drug-related problems, rendering it possible to assess therapy appropriateness. Accordingly, an accurate medication history is crucial in ensuring correct medication prescriptions during hospital stay and upon discharge [6]. Conversely, medication history errors may result in iatrogenic patient harm, leading to prolonged length of stay or even death [7-9]. Obtaining an accurate medication history early on during hospitalization, for example, while the patient is still in the ED, is clearly the preferred approach. Fixing medication errors downstream has largely been considered to be both unsafe and inefficient [10].

However, the medication reconciliation process is prone to many errors and is labor-intensive [10]. The hectic environment along with competing medical priorities among physicians and nurses, unreliable information due to the acute illness of the patient, and the nonfamiliarity of drug names for the patient or family members lead to incomplete and inaccurate medication histories [11,12]. The MARQUIS study found that pharmacist involvement was a key component in improving the reliability of medication reconciliation [13]. Unfortunately, this requires a level of staffing resources beyond usual care, which is often not available [10,14]. As a result, many patients simply do not receive a thorough and complete medication reconciliation. Therefore, an approach to prioritize patients who are most likely to benefit from medication reconciliation would be of great benefit.

To date, literature on medication reconciliation risk stratification tools is scarce [1,15-18]. Our research group published a prediction model almost a decade ago that identified patients at risk for medication discrepancies upon ED presentation. The model was based on 13 parameters that can be easily extracted from the electronic health record (EHR). The downside of this prediction model was its inability to identify clinically relevant discrepancies, thereby limiting the usefulness of this model [19].

Hence, we need an approach enabling us to identify those patients at the ED who are at increased risk for clinically relevant discrepancies. As such, priority for a pharmacist-led medication reconciliation can be given to these patients. The aim of this study was to develop and externally validate a prediction model, the MED-REC predictor, that can be programmed in the EHR to identify patients at risk for at least 1 clinically relevant medication discrepancy upon ED presentation.

## Methods

Appropriate reporting of our prediction model was performed based on the TRIPOD (Transparent Reporting of a Multivariable Prediction Model for Individual Prognosis or Diagnosis) guideline [20].

### Study Design and Setting

A prospective, multicenter study was carried out at the EDs of University Hospitals Leuven (UZ Leuven) and General Hospital Sint-Jan Brugge-Oostende AV (AZ Sint-Jan Brugge-Oostende), Belgium, from November 2017 to May 2022.

UZ Leuven is a tertiary 1995-bed teaching hospital. The ED consists of an admission and a treatment area (12 boxes), an ambulatory zone, a pediatric zone, and 3 observation care units (30 beds of which 6 are equipped as intensive care beds). On average, 197 patients per day visit the ED, of which approximately 30% (60/197) are hospitalized.

AZ Sint-Jan Brugge-Oostende is a 1182-bed general hospital. The ED consists of an admission and treatment area (11 boxes), an ambulatory zone, a pediatric zone, and 3 beds reserved for critically ill patients. Approximately 92 patients per day visit the ED, of which 38% (35/92) are hospitalized.

Depending on the presumed medical diagnosis, patients are treated by a physician specialized in internal medicine, surgery, emergency medicine, pediatrics, or psychiatry. As part of the obligatory patient assessment, a medication history is obtained by the physician in all patients within 24 hours of the ED visit and is entered in a dedicated medication history module in the EHR. Subsequently, the entered medication history should be electronically validated once considered complete.

### Patients

Pharmacy, biomedical sciences, or hospital pharmacy students trained in medication reconciliation enrolled planned admission patients on weekdays between 8:30 AM and 5 PM, starting with the patient present the longest and proceeding in descending order. Medication reconciliation was performed based on student availability during their internship. Medication reconciliation was performed independently from the information obtained by physicians. While medication reconciliation was performed between 8:30 AM and 5 PM on weekdays, the sample included patients arriving to the ED during the evening, night, and weekend as a result of the need for observation in the ED or waiting for inpatient beds. Exclusion criteria were (1) patients aged younger than 18 years; (2) patients receiving end-of-life care; (3) patients transferred from another hospital or ward; (4) patients not speaking Dutch, French, or English; (5) patients discharged home or deceased before the medication history was obtained; (6) patients with intentional intoxication; or (7) patients in isolation due to a possible COVID-19 infection.

### Training

Every student performing medication reconciliation received formal training by a clinical pharmacist (SDW or GVDS) prior to patient recruitment. Interrater reliability for medication reconciliation was evaluated using a similar methodology as previously described by Pippins et al [1]. Every student had to obtain 4 fictional medication histories through role-play. Results were compared with the best possible medication history as composed by the research team. A cutoff of 90% agreement was set for each fictive case.

### Data Collection

#### Overview

Three distinct datasets were collected. The first dataset (UZ Leuven; from November 2017 to September 2019) was used to develop and internally validate the MED-REC predictor. The second dataset (UZ Leuven; from October 2021 to April 2022) and the third dataset (AZ Sint-Jan Brugge-Oostende; from April to May 2022) were used for temporal and geographic external validation, respectively. Temporal validation assesses the performance of the model on patients from a later period, and geographic validation uses data from a different hospital [20].

#### Medication Reconciliation

A structured form—including a checklist, a table, and a standardized list of questions—and a protocol were used to guide the students in obtaining the best possible medication history (Multimedia Appendix 1) [19]. The protocol included 3 steps. The first step consisted of a conversation with the patient. The patient was explicitly asked about prescription and nonprescription drugs easily forgotten, such as transdermal patches, inhalers, and dietary supplements. If the patient was unable to communicate or was unaware of his or her current medication intake, a close relative was interviewed. Other sources were used as well to retrieve information including verification of medication boxes if brought to the ED, previous medical records, and referral notes. The second step consisted of contacting the community pharmacist. The third step involved contacting the general practitioner if there was a discrepancy between the list obtained from the patient or his or her relative and the list obtained from the community pharmacist, or if the community pharmacist had no overview of the patient’s current medication intake.

When resources were insufficient to verify the medication schedule, for example, when neither the community pharmacist nor the general practitioner could be reached and the gold standard was thus unavailable, the patient was excluded.

#### Discrepancies and Their Clinical Relevance

The medication history obtained by the trained student was defined as the gold standard. A discrepancy was defined as any difference between the physician-acquired medication history and the gold standard. Discrepancies consisted of a drug omission or commission, an incorrect dose or dosage regimen.

A classification was developed to categorize discrepancies according to perceived clinical relevance. The classification was defined by an expert panel, consisting of 4 clinical pharmacists (SDW, LVdL, ES, and IS) and 1 physician (PV) with substantial expertise in medication management. SDW and LVdL are experienced ED clinical pharmacists, ES is a junior clinical pharmacist, and IS is a senior clinical pharmacist. PV is a senior physician specializing in both internal medicine as well as emergency medicine, with 20 years of experience. The expert panel independently assigned clinical relevance categories to the major drug classes listed in the national (Belgian) drug formulary. In case of the absence of consensus, SDW made the final decision. Three categories were used, which were based on the methodology proposed by Cornish et al [2]. Only discrepancies that may lead to severe discomfort or clinical deterioration were included for the development of the MED-REC predictor and are further specified in this work as “clinically relevant discrepancies.” An overview is presented in Table 1. Discrepancies involving one of these drugs are assumed to affect treatment or urgent procedures in the ED and should be corrected before the patient is discharged from the ED. “As needed” medication was never considered clinically relevant.

**Table 1:** Classification of clinically relevant medication discrepancies in medication histories. A drug omission or commission and an incorrect dose or dosage regimen concerning these drugs were considered clinically relevant discrepancies. Groups were based on the World Health Organization’s Anatomical Therapeutic Chemical (ATC) classification system.

Anatomical or pharmacological group	Chemical, pharmacological, or therapeutic subgroup
A. Alimentary tract and metabolism	A10 drugs used in diabetes
B. Blood and blood forming organs	B01A antithrombotic agents B02 antihemorrhagics
C. Cardiovascular system	C01 cardiac therapy C02 antihypertensives C03 diuretics C04 peripheral vasodilators C07 beta blocking agents C08 calcium channel blockers C09 agents acting on the renin-angiotensin system
H. Systemic hormonal preparations. (excluding sex hormones and insulins)	H01 pituitary and hypothalamic hormones and analogs H02 corticosteroids for systemic use
J. Anti-infectives for systemic use	J01 antibacterials for systemic use J02 antimycotics for systemic use J04 antimycobacterials J05 antivirals for systemic use J06 immune sera and immunoglobulins
L. Antineoplastic and immunomodulating agents	L01 antineoplastic agents L03 immunostimulants L04 immunosuppressants
N. Nervous system	N02A opioids N03 antiepileptics N05 psycholeptics N07A parasympathomimetics N07B drugs used in addictive disorders N07X other nervous system drugs
P. Antiparasitic products, insecticides, and repellents	P01 antiprotozoals
R. Respiratory system	R03 drugs for obstructive airway disease

#### Patient-, Drug-, ED Visit–, and Medication Reconciliation–Related Factors

A structured form was used to collect patient-, drug-, ED visit–, and medication reconciliation–related factors. Details are provided in Multimedia Appendix 2.

### Model Development

Variables were considered for the MED-REC predictor if (1) a significant association between the variable and the occurrence of medication discrepancies had been shown in the literature [21] or if (2) the variable was considered of interest as discussed by our multidisciplinary research team. Furthermore, EHR availability of the variable upon ED visit was required.

Multivariable logistic regression with backward stepwise selection according to the Akaike information criterion was used to select the final model. The presence of at least 1 clinically relevant discrepancy was the dependent variable. Sex, age, language (Dutch, French, or English), residence before ED admission (home, nursing home, or others), transport to the ED (patient’s own transport, ambulance, or emergency physician transport vehicle), ED triage acuity scale using the Emergency Severity Index (ESI), number of drugs documented by the ED physician in the medication history module, number of drugs documented by the ED physician in the medication history module of a certain first WHO (World Health Organization) Anatomical Therapeutic Chemical (ATC) level (ATC A, ATC B, etc), number of high-risk drugs documented by the ED physician in the medication history module, time of ED visit (morning, afternoon, evening, or night), specialty of the physician obtaining the medication history (emergency medicine, internal medicine, psychiatry, or surgery), availability of a regionally shared electronic medication record, and electronic validation of the medication history were investigated as predictor variables.

Continuous predictors were evaluated for nonlinearity using scatter plots, and various transformations were applied to address any nonlinear relationships. However, these transformations did not improve model performance compared with simpler models. Therefore, we decided to forego transforming continuous predictors to maintain interpretability and simplicity.

Missing data were present for the ED triage acuity scale (160/824, 19.4%) and were handled using dummy variable adjustment.

### Internal and External Validation

The predictive performance of the model was assessed by measuring calibration, discrimination, and classification in all datasets. Calibration reflects the agreement between model-based predictions and observed outcomes, and was assessed using calibration plots with a target slope and intercept of 1 and 0, respectively. Discrimination refers to the ability of the prediction model to differentiate between those experiencing at least 1 clinically relevant discrepancy and those who do not. Discrimination was evaluated using the concordance index (*c*-index), for example, the area under the receiver operating characteristic curve (AUROC) for models with binary end points. Bootstrap 95% CIs were calculated. We assessed the following classification measures in various scenarios relevant to daily clinical practice: specificity, sensitivity, positive predictive value, negative predictive value, positive likelihood ratio, negative likelihood ratio, and alert rate. Scenarios were defined by specific probability thresholds, which were used to classify patients predicted to show at least 1 clinically relevant medication discrepancy or not. We investigated the Youden index (maximized sensitivity and specificity), with a sensitivity of at least 80% for hospitals with sufficient staffing resources and a specificity of at least 80% for hospitals with limited staffing resources.

### Model Update

Commonly, the development and validation datasets differ in proportion to outcome events, yielding poor calibration of the original model when applied to new data. Accordingly, adjusting the intercept of the original model to the validation sample can improve calibration [20]. In such a model update, the correction factor for the intercept is estimated in the validation dataset and should be added to the intercept of the original model when applying the model to new patients [22].

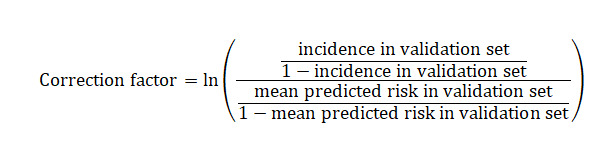



### Decision Curve Analysis

The utility of the model for decision-making was evaluated using decision curve analysis [23-25]. In brief, decision curve analysis calculates a “net benefit” for a prediction model by comparing it to (at least) 2 default strategies: exposing either all patients or no patients to an intervention—in this study, medication reconciliation. The net benefit weighs the true positives against the false positives and is calculated using the following equation [23]:

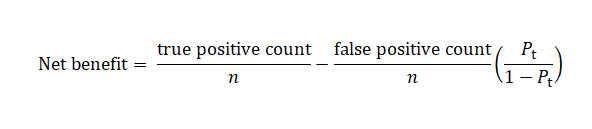



Where:

- *n* is the total number of patients
- *P*_t_ is the threshold probability, which reflects the probability at which a health care provider would choose to perform a medication reconciliation to detect clinically relevant medication discrepancies.
- true positive count/*n* represents the proportion of patients correctly identified as needing a medication reconciliation.
- false positive count/*n* represents the proportion of patients incorrectly identified as needing a medication reconciliation
- *P*_t_/(1–*P*_t_) is given a weighting factor based on the threshold probability *P*_t_. It adjusts the impact of false positives by reflecting how tolerable the false positives are relative to the true positives.
  For instance, a higher *P*_t_—and hence higher *P*_t_/(1–*P*_t_)—increases the importance of false positives in estimating the net benefit of a certain strategy.
  Conversely, when assuming a lower *P*_t_, the importance of false positives decreases, putting more emphasis on identifying true positives.

The net benefit is plotted across a range of threshold probabilities, generating the “decision curve.”

### Comparison of the MED-REC Predictor With Existing Selection Algorithms

The MED-REC predictor was compared with two existing patient selection algorithms, as used in practice by ED pharmacists and physicians: (1) random selection and (2) patients aged ≥75 years and taking ≥5 drugs.

### Statistical Analysis

Data analysis was performed using R (version 4.1.1; R Core Team). The R packages *givitiR*, *pROC* [26], and *dcurves* were used to assess calibration, discrimination, and net benefit, respectively. Descriptive statistics were presented as frequency with percentage for categorical data and as median with IQR for continuous data. Baseline characteristics of the 3 cohorts were compared by chi-square test for categorical data and by the Kruskal-Wallis test for continuous data.

No sample size calculation was performed for model development as we anticipated a large number of inclusions and a high number of discrepancies [19]. A sample size calculation was performed for the temporal validation to estimate the absolute agreement rate with a prespecified precision, defined as the maximum width of the associated 95% CI. In this calculation, we assumed an absolute agreement rate of 70% between the MED-RED predictor and the gold standard, to be estimated with a 95% CI that should have a maximal half-width of 5%. The required sample size was estimated at 333 patients, which also exceeded the minimum of 100 events and nonevents as suggested by the TRIPOD guidelines, assuming a prevalence of 35% as observed in the development dataset [20]. For the geographic validation, we intended to include a pragmatic sample of 100 patients, based on resources available at the external hospital.

### Ethical Considerations

The study was approved by the Ethics Committee Research UZ/KU Leuven and AZ Sint-Jan Brugge Oostende (S60638). Written informed consent was obtained prior to inclusion from each patient or relative when the patient was deemed incapacitated. All study data were deidentified. Participation was voluntary and not financially compensated.

## Results

### Overview

**Figure 1:**
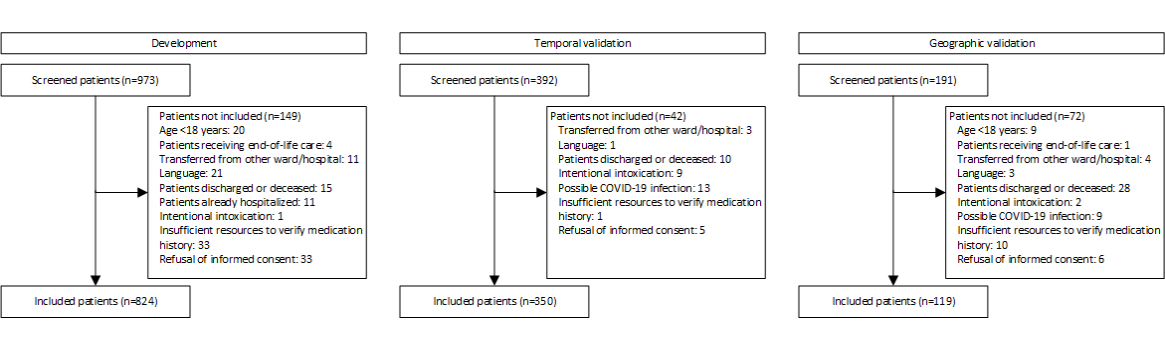
Study flow diagram.

In total, 824, 350, and 119 patients were included in the development, temporal validation, and geographic validation dataset, respectively. A flowchart of the study participants is presented in Figure 1. Patient characteristics, drug-related factors, and factors related to ED visits and the medication reconciliation process that were considered candidate variables for the MED-REC predictor are presented in Table 2. Data on the number of drugs before and after medication reconciliation and data on observed medication discrepancies are shown in Table 3. All students achieved the cutoff level of interrater reliability with a mean percent agreement of 96%.

**Table 2:** Description of the development and external validation cohorts. Baseline characteristics were compared by the chi-square test for categorical data and by the Kruskal-Wallis test for continuous data.

				Development (n=824)		Temporal validation (n=350)		Geographic validation (n=119)		*P* value
**Study period**				2017-2019		2021-2022		2022		—^a^
**Patient-related factors**										
	**Sex, n (%)**									
		Female	385 (46.7)		199 (56.9)		66 (55.5)		.003	
	**Age (years), median (IQR)**			69 (55-80)		67 (55-79)		69 (55-78)		.91
	**Language, n (%)**									.68
		Dutch	810 (98.3)		347 (99.1)		118 (99.2)			
		French	11 (1.3)		3 (0.9)		1 (0.8)			
		English	3 (0.4)		0 (0)		0 (0)			
	**Residence before admission, n (%)**									.37
		Home	798 (95.8)		341 (97.4)		116 (97.5)			
		Nursing home	29 (3.5)		9 (2.6)		2 (1.7)			
		Other	6 (0.7)		0 (0)		1 (0.8)			
	**ESI^b^triage score, n (%)**									<.001
		1	1 (0.1)		5 (1.4)		0 (0)			
		2	117 (14.2)		102 (29.1)		14 (11.8)			
		3	175 (21.2)		116 (33.1)		91 (76.5)			
		4	14 (21.7)		19 (5.4)		14 (11.8)			
		5	3 (0.4)		1 (0.3)		0 (0)			
		Unknown	75 (9.1)		107 (30.6)		0 (0)			
	**Transport to the ED^c^, n (%)**									<.001
		Patient’s own transport	706 (85.7)		243 (69.4)		76 (63.9)			
		Ambulance	97 (11.8)		80 (22.9)		25 (21.0)			
		Emergency physician vehicle transport	21 (2.5)		27 (7.7)		18 (15.1)			
**Drug-related factors**										
	**Number of medications reported by the ED physician per patient at time of ED visit, median (IQR)**			6 (3-10)		5 (3-9)		6 (3-9)		.23
	**Number of medications reported by the ED physician per patient at time of ED visit by first-level ATC^d^ group, median (IQR)**									
		ATC A drugs (alimentary tract and metabolism)	1 (0-2)		1 (0-3)		1 (0-2)		.74	
		ATC B (blood and blood forming agents)	1 (0-1)		1 (0-1)		0 (0-1)		.18	
		ATC C (cardiovascular system)	1 (0-3)		1 (0-2)		1 (0-2)		.88	
		ATC D (dermatologicals)	0 (0-0)		0 (0-0)		0 (0-0)		.08	
		ATC G (genito urinary system and sex hormones)	0 (0-0)		0 (0-0)		0 (0-0)		.34	
		ATC H (systemic hormonal preparations, excl. sex hormones and insulins)	0 (0-0)		0 (0-0)		0 (0-0)		.57	
		ATC J (anti-infectives for systemic use)	0 (0-0)		0 (0-0)		0 (0-0)		.004	
		ATC L (antineoplastic and immunomodulating agents)	0 (0-0)		0 (0-0)		0 (0-0)		.60	
		ATC M (musculo-skeletal system)	0 (0-0)		0 (0-0)		0 (0-0)		.04	
		ATC N (nervous system)	1 (0-2)		1 (0-2)		1 (0-2)		.28	
		ATC P (antiparasitic products, insecticides, and repellents)	0 (0-0)		0 (0-0)		0 (0-0)		.20	
		ATC R (respiratory system)	0 (0-0)		0 (0-0)		0 (0-0)		.72	
		ATC S (sensory organs)	0 (0-0)		0 (0-0)		0 (0-0)		.007	
		ATC V (various)	0 (0-0)		0 (0-0)		0 (0-0)		.39	
**ED visit related factors**										
	**Specialty of the ED physician, n (%)**									<.001
		Emergency medicine	107 (13.0)		61 (17.4)		19 (16.0)			
		Internal medicine	569 (69.0)		254 (72.6)		77 (64.7)			
		Surgery	148 (18.0)		35 (10)		21 (17.6)			
		Psychiatry	0 (0)		0 (0)		2 (1.7)			
	**Time of ED visit, n (%)**									<.001
		Morning	340 (41.3)		157 (44.9)		81 (68.0)			
		Afternoon	137 (16.6)		33 (9.4)		16 (13.4)			
		Evening	260 (31.5)		60 (17.1)		1 (0.8)			
		Night	87 (10.5)		100 (28.6)		21 (17.6)			
**Medication reconciliation-related factors, n (%)**										
	Availability of regional shared electronic medication record (yes)			111 (13.5)		80 (22.9)		46 (38.6)		<.001
	Electronic validation of the medication history by the ED physician (yes)			155 (18.8)		63 (18.0)		31 (26.0)		.14
**Outcome, n (%)**										
	At least 1 clinically relevant medication discrepancy, (yes)			287 (35)		131 (37)		58 (49)		.01

^a^Not applicable.

^b^ESI: Emergency Severity Index.

^c^ED: emergency department.

^d^ATC: Anatomical Therapeutic Chemical.

**Table 3:** The number of drugs before and after medication reconciliation and observed medication discrepancies in the development and external validation cohorts.

	Development (n=824)	Temporal validation (n=350)	Geographic validation (n=119)
Total number of drugs reported by the ED^a^ physician, n	5486	2266	734
Number of drugs reported by the ED physician per patient, median (IQR)	6 (3-10)	5 (3-9)	6 (3-9)
Total number of drugs after medication reconciliation	6456	2614	926
Number of drugs per patient after medication reconciliation, median (IQR)	7 (4-11)	7 (4-10)	8 (4-10)
Total number of discrepancies, n	1604	801	451
Number of discrepancies per patient, median (IQR)	1 (0-3)	2 (1-3)	3 (2-5)
Total number of clinically relevant discrepancies, n	475	205	116
Number of clinically relevant discrepancies per patient, median (IQR)	0 (0-1)	0 (0-1)	1 (0-2)
At least 1 clinically relevant discrepancy (yes), n (%)	287 (35)	131 (37)	58 (49)

^a^ED: emergency department.

### Model Development

Out of the 824 included patients, 287 (35%) had at least 1 clinically relevant discrepancy, with a total of 475 clinically relevant discrepancies.

**Figure 2:**
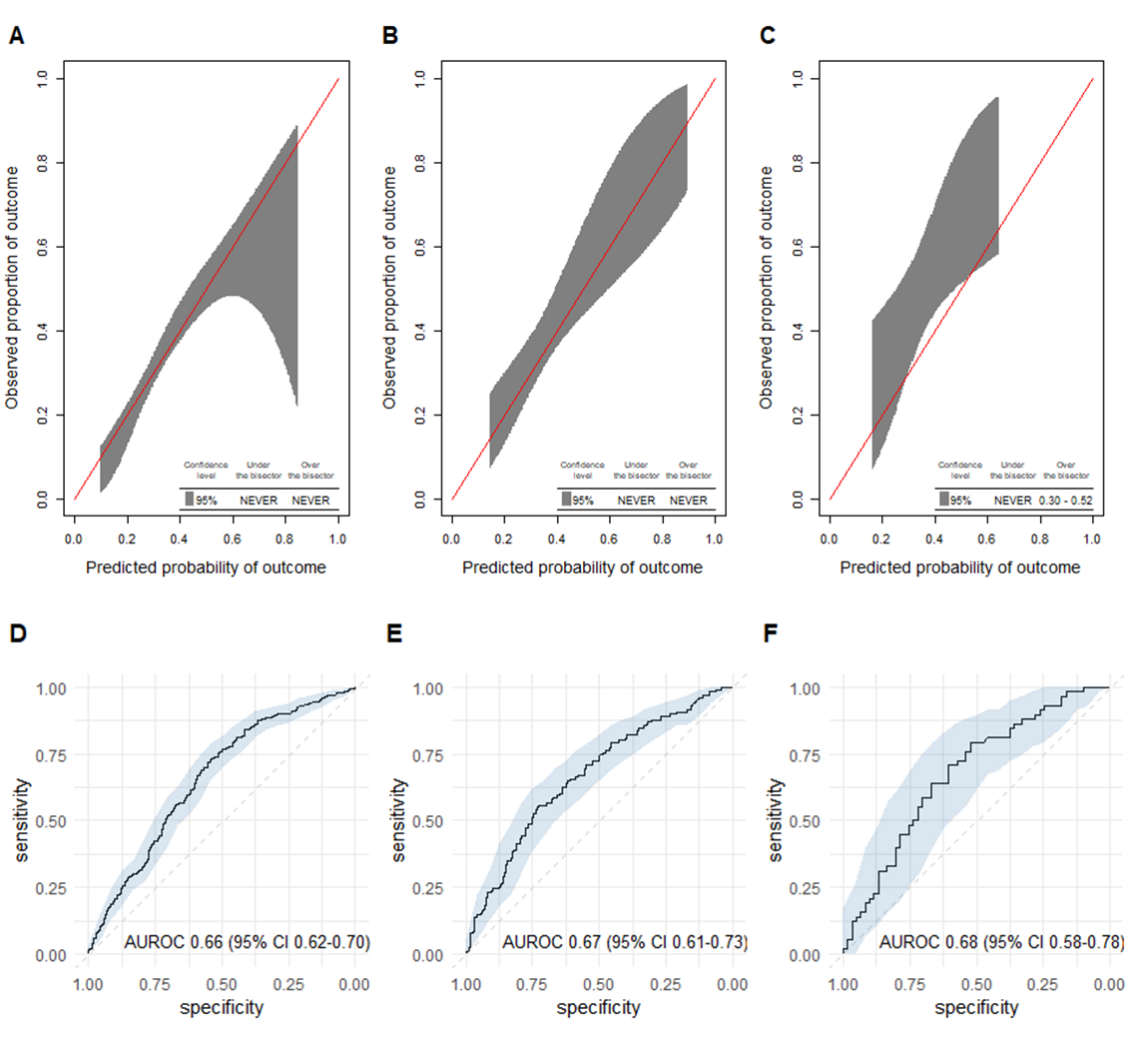
Calibration plots for the MED-REC predictor in the (A) development, (B) temporal validation, and (C) and geographic validation dataset, as well as AUROC curves for the MED-REC predictor in the (D) development, (E) temporal validation, (F) and geographic validation dataset. The red line represents perfect calibration. The shaded area represents the 95% CI. AUROC: area under the receiver operating characteristic curve.

After feature selection based on 26 initial variables, the final prediction model contained 8 variables (Table 4). The retained predictor variables were found in similar proportions across all datasets (Table 2). Figure 2A shows that our MED-REC predictor was well calibrated over a broad range of probabilities. The calibration slope was 0.72 and the intercept was 0.03. Discrimination was moderate with an AUROC of 0.66 (95% CI 0.62-0.70; Figure 2D). Classification measures are shown in Table 5. The Youden index, a sensitivity of at least 80%, and a specificity of at least 80% were found at probability thresholds of 0.31, 0.25, and 0.45, respectively.

**Table 4:** Equation of the MED-REC predictor estimating the probability of having at least 1 clinically relevant discrepancy. The shape of the formula is 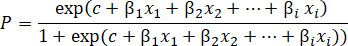, where P is the probability of having at least 1 clinically relevant discrepancy, c is the intercept, xi is the predictor variable and βi is the corresponding β coefficient. For each predictor variable, the β coefficient, odds ratio, 95% CI, and the *P* value are presented.

Variable			Coefficient		OR^a^ (95% CI)		*P* value
**Intercept**			–1.82		0.16 (0.08 to 0.30)		<.001
**Age (years)**			0.012		1.01 (1.00 to 1.02)		.02
**Residence of the patient before admission (reference category: home)**							
	Residence of the patient (nursing home)	–0.96		0.38 (0.15 to 0.90)		.03	
	Residence of the patient (other)	–0.79		0.45 (0.06 to 2.25)		.36	
	Number of drugs reported by the ED^b^ physician	–0.094		0.91 (0.83 to 0.99)		.04	
	Number of “ATC A”^c^ drugs reported by the ED physician (alimentary tract and metabolism)	0.276		1.32 (1.13 to 1.55)		<.001	
	Number of “ATC C” drugs reported by the ED physician (cardiovascular system)	0.167		1.18 (1.02 to 1.37)		.03	
	Number of “ATC N” drugs reported by the ED physician (nervous system)	0.206		1.23 (1.06 to 1.42)		.005	
	Number of “ATC P” drugs reported by the ED physician (antiparasitic products insecticides and repellents)	1.712		5.54 (1.07 to 41.4)		.05	
	Number of “ATC R” drugs reported by the ED physician (respiratory system)	0.269		1.31 (1.09 to 1.58)		.004	

^a^OR: odds ratio.

^b^ED: emergency department.

^c^ATC: Anatomical Therapeutic Chemical.

**Table 5:** Classification measures in each cohort using 3 different probability thresholds (0.25, 0.31, and 0.45). The output of the logistic regression model is a score between 0 and 1. To make a binary classification decision, a probability threshold is applied. If the calculated probability exceeds this threshold, it is categorized as having at least 1 clinically relevant discrepancy. The probability threshold is a crucial parameter that affects the trade-off between sensitivity and specificity.

Datasets and probability threshold			Specificity (%)	Sensitivity (%)	PPV^a^ (%)		NPV^b^ (%)	LR+^c^		LR–^d^		Alert rate (%)
**Development dataset**												
	0.25	26		90	40	84		1.23	0.37		79	
	0.31	54		74	46	78		1.61	0.48		56	
	0.45	84		29	49	69		1.83	0.85		20	
**Temporal validation dataset**												
	0.25	26		89	42	80		1.20	0.42		80	
	0.31	51		73	47	76		1.48	0.54		58	
	0.45	88		25	55	66		2.04	0.85		17	
**Geographic validation dataset**												
	0.25	26		88	53	70		1.19	0.46		81	
	0.31	49		79	60	71		1.56	0.42		65	
	0.45	87		24	64	55		1.84	0.87		18	

^a^PPV: positive predictive value.

^b^NPV: negative predictive value.

^c^LR+: positive likelihood ratio.

^d^LR–: negative likelihood ratio.

### Temporal Validation

Out of 350 included patients, 131 (37%) had at least 1 clinically relevant discrepancy, with a total of 205 clinically relevant discrepancies. Excellent calibration was found over the whole range of probabilities with a slope of 1.09 and an intercept of 0.18 (Figure 2B). Discrimination was moderate with an AUROC of 0.67 (95% CI 0.61-0.73), which was not different compared with the development dataset (Figure 2E). Classification measures are shown in Table 5.

### Geographic Validation

Out of 119 included patients, 58 (49%) had at least 1 clinically relevant discrepancy, with a total of 116 clinically relevant discrepancies. The calibration curve is shown in Figure 2C. A slope of 1.35 and an intercept of 0.83 were found. The predictions underestimated the observed outcome probability of having at least 1 clinically relevant medication discrepancy for estimated probabilities between 0.3 and 0.52. Discrimination was moderate with an AUROC of 0.68 (95% CI 0.58-0.78), which was not different compared with the development dataset (Figure 2F). Classification measures are shown in Table 5.

### Model Update

**Figure 3:**
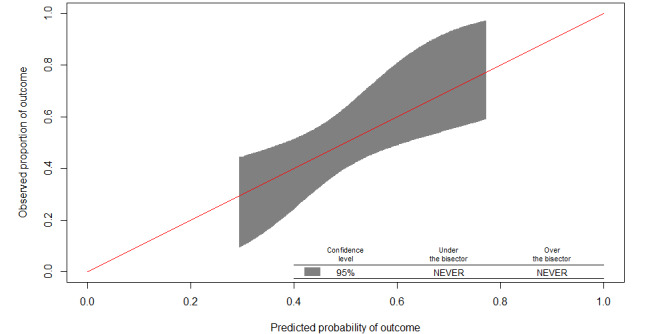
Calibration plot for the updated MED-REC predictor in the geographic validation dataset. The red line represents perfect calibration. The shaded area represents the 95% CI.

The incidence in the geographic validation dataset was 49% and the mean predicted risk was 35%. Adjustment of the intercept was performed and calibration and discrimination were reassessed. The correction factor was 0.58. The calibration plot of the updated model in the geographic validation dataset showed excellent calibration over the whole range of probabilities (Figure 3). Discrimination of the updated model did not differ from that of the original model (AUROC 0.68).

### Decision Curve Analysis

**Figure 4:**
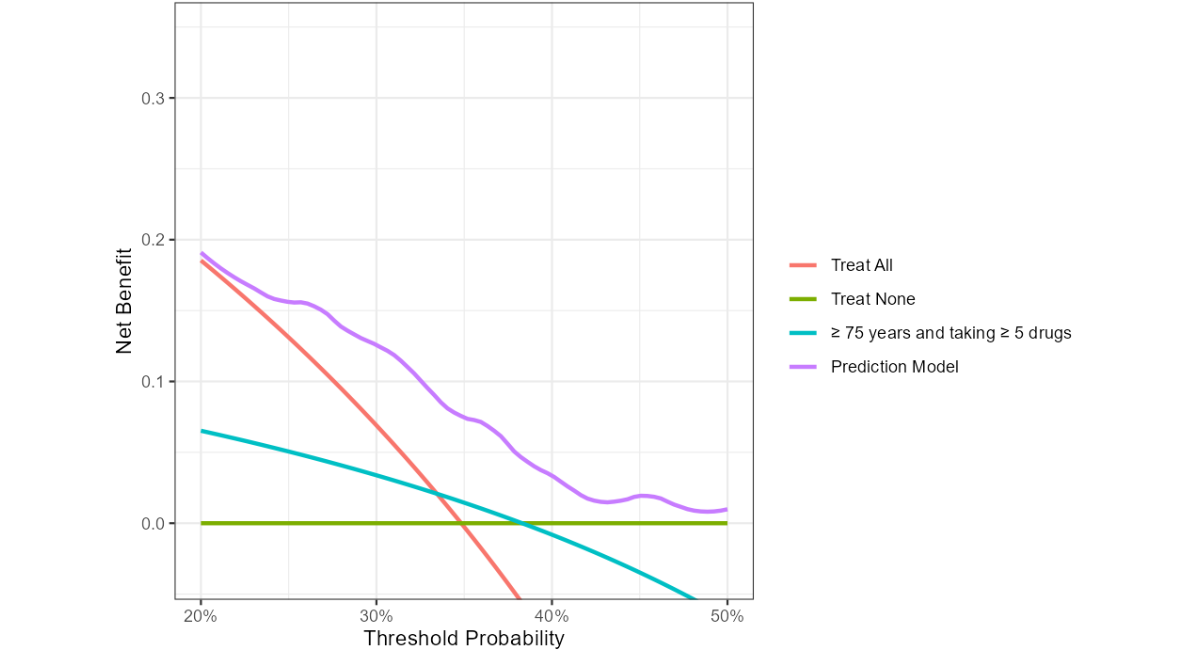
Decision curve analysis for the MED-REC predictor in the development dataset. Red line (treat all): all patients receive medication reconciliation. Green line (treat none): none of the patients receives medication reconciliation. Blue line: selection of patients who are ≥75 years and take ≥5 drugs. Purple line: MED-REC predictor. The MED-REC predictor shows net benefit over the range of clinically reasonable threshold probabilities. Classifying a patient as having at least 1 clinically relevant medication discrepancy will trigger performing a comprehensive medication reconciliation. Accordingly, lower threshold probabilities are preferred if one is worried about missing clinically relevant discrepancies. Higher threshold probabilities are preferred if one is worried about additional costs or resources associated with medication reconciliation. For instance, a probability threshold of 20% implies that you expect to find at least 1 clinically relevant discrepancy in 1 out of 5 reconciled patients.

The MED-REC predictor showed net benefit over the other strategies (treat all, treat none, and patients that are ≥75 years and taking ≥5 drugs) over a range of clinically reasonable threshold probabilities (20%-50%) in the development cohort (Figure 4). Decision curve analysis confirmed the net benefit of the MED-REC predictor over a similar range of threshold probabilities in the temporal validation cohort (Figure S1 in Multimedia Appendix 3). In the geographic validation cohort, the net benefit for the updated MED-REC predictor was demonstrated at higher threshold probabilities, starting from 30% (Figure S2 in Multimedia Appendix 3). The miscalibrated MED-REC predictor did not show a net benefit (Figure S3 in Multimedia Appendix 3).

### Comparison of the MED-REC Predictor With Existing Selection Algorithms

#### Random Selection

**Figure 5:**
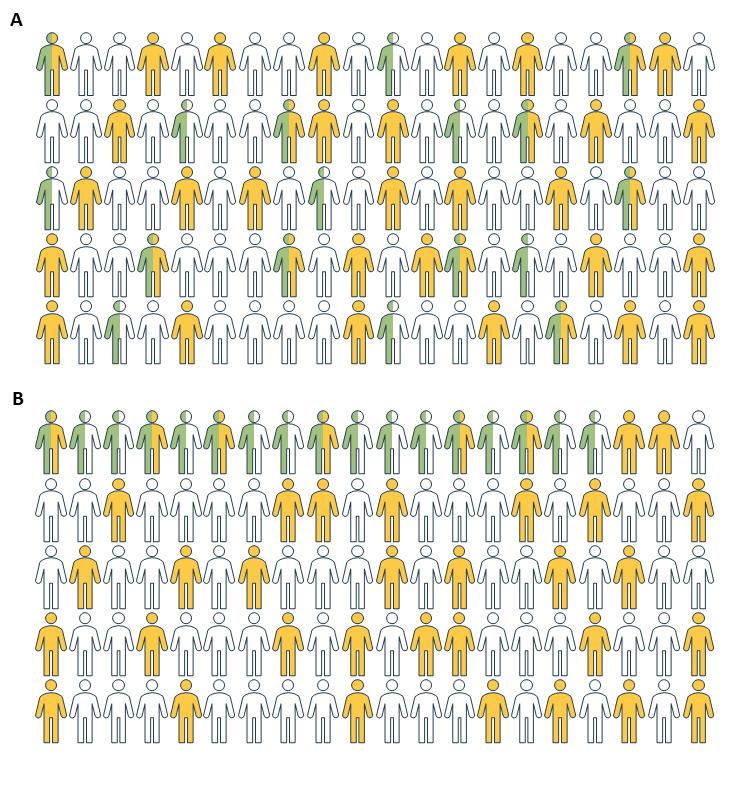
Comparison of the (A) MED-REC predictor to (B) random selection. Calculations are based on the classification measures of the temporal validation. A probability threshold of 0.45 was used, considering limited staffing resources for medication reconciliation in our hospitals. Patients with at least 1 clinically relevant discrepancy are presented in yellow. Patients without clinically relevant discrepancies are presented in white. Medication reconciliation is performed for patients in green. (A) If the MED-REC predictor is applied to 100 patients, 17 will be identified as high risk, alerting the pharmacist to perform a medication reconciliation. For 9 of these patients (17×0.55), at least 1 clinically relevant medication discrepancy will be found. (B) When a medication reconciliation is performed for 17 randomly selected patients, at least 1 clinically relevant medication discrepancy will be found in 6 patients (17×0.37). Eleven patients, as opposed to 8 with the MED-REC predictor, would not have needed the pharmacy staff’s intervention.

A comparison of the MED-REC predictor to random selection is presented in Figure 5.

#### Patients Aged ≥75 Years and Taking ≥5 Drugs

The AUROC curves for this selection algorithm showed no discrimination across the 3 datasets. The AUROCs were 0.52 (95% CI 0.48-0.55), 0.58 (95% CI 0.53-0.63), and 0.54 (95% CI 0.46-0.63) for the development, temporal validation, and geographic validation dataset, respectively. The MED-REC predictor discriminated significantly better across the 3 datasets (*P*<.001, *P*=.002, and *P*=.003, respectively). Additionally, the net benefit analysis indicated that the MED-REC predictor provided the highest net benefit (Figure 4).

## Discussion

### Principal Findings

We developed and externally validated the MED-REC predictor, according to the current standards of predictive modeling [20]. The final model contains 8 variables that can be easily extracted from the EHR upon ED presentation. The MED-REC predictor identifies patients at risk for at least 1 clinically relevant discrepancy with moderate performance and clearly outperforms existing selection strategies. The clinical usefulness of the model was demonstrated through decision curve analysis, which showed net benefit for the MED-REC predictor compared with default strategies and typical selection criteria. While there is no question that conducting a comprehensive medication reconciliation for each hospital admission is of the utmost importance, the stark reality is that this objective is frequently untenable in clinical practice. Hence, our MED-REC predictor can be used to guide the rational use of limited resources at the ED and is especially useful in countries where resources are insufficient.

As an outcome variable, we opted for clinically relevant discrepancies, allowing us to focus on patients with the highest need for medication reconciliation. The current prediction model contrasts with our previously developed model, which screened for any medication discrepancy [19]. After applying the former model in clinical practice, we experienced its lack of clinical usefulness as the model identified many patients with nonclinically relevant discrepancies, for example, discrepancies concerning vitamin supplements.

We validated the MED-REC predictor in both a temporal and geographic validation dataset. Discrimination was moderate and was retained in both external validation datasets, demonstrating the robustness of our MED-REC predictor. The lack of excellent discrimination might be due to other unknown or unmeasured variables not included in our model. However, upon ED presentation, readily available patient information is limited. Calibration was excellent in the temporal validation dataset, whereas underestimation of the observed outcome by the model predictions was observed in the geographic validation dataset, due to a higher prevalence of the outcome in the geographic validation dataset. A simple model update improved calibration in this setting [22,27].

Net benefit was demonstrated over a range of clinically reasonable threshold probabilities (20%-50%) in both the development and temporal validation datasets. Notably, in the geographic validation dataset, the net benefit was observed starting from a higher probability threshold of 30%, which can be attributed to the higher prevalence of the outcome in this cohort (Figure S2 in Multimedia Appendix 3).

Implementation of the MED-REC predictor, a relatively simple prediction model based on readily available variables, is feasible in clinical practice. A precondition is the structural availability of the variables in the EHR in order for the MED-REC predictor to work optimally. The probability threshold can be easily customized to accommodate the available staffing resources. A lower probability threshold will lead to a higher alert rate and fewer false negative results, which might be interesting for hospitals with a large staff available to perform medication reconciliation. A higher probability threshold will be better suited for hospitals with limited staff, hence decreasing the false positive results. The decision curve analysis illustrates how the probability threshold impacts clinical decision-making (Figure 4). The threshold reflects the trade-off between the benefits of true positives and the potential harms of false positives. For instance, a probability threshold of 20% suggests a willingness to perform medication reconciliation on 5 patients to identify the outcome, at least 1 clinically relevant discrepancy, in one patient. This threshold implies that the benefit of detecting one true positive is deemed 4 times greater than the potential (financial) harm of performing unnecessary medication reconciliations [25].

Literature on medication reconciliation risk stratification tools is scarce. Moreover, most published models are not validated at all or are limited to discrimination measurement. Pippins et al [1] published the potential adverse drug event tool based on an observational study on general medical wards. An exploratory model was developed to predict the number of potential adverse drug events, but this model was not validated. Besides, automatization of their tool is limited because not all included predictors are structurally available in the EHR, for example, patient understanding of preadmission medications, and family members or caregivers as the source of preadmission medication information. Ebbens et al [16] developed and validated a risk prediction model to identify patients at risk for medication discrepancies at planned hospital admission. Retained variables were the number of drugs and the presence of cardiovascular and respiratory comorbidities. Good discrimination was found in the development cohort (AUROC 0.75) but was not retained in the external validation cohort (AUROC 0.54). Unfortunately, calibration was not assessed. Recently, Chu et al [15] reported a case series on the implementation of medication reconciliation prediction models in clinical practice. These models were initially developed to identify medication errors upon discharge as part of the MARQUIS2 [28] study and were adapted by removing variables that are not available upon ED presentation. However, the models were not developed based on predictive modeling standards nor was their performance assessed to account for this new setting. Thus, we cannot draw any conclusions regarding the robustness or generalizability of these models. Similar to our study, Damlien et al [17] developed and tested a prediction model to identify patients at risk for clinically relevant medication discrepancies upon ED admission. The authors included a limited sample of 276 patients over the course of 7 weeks, which was used for both development and validation of their model. Four variables were included, for example, sex, age ≥60 years, hospital admission in the last 12 months, and admission reason (malfunction, surgical, or cancer). Using a fixed cutoff value, the model classified patients as having a high versus low risk for discrepancies. An AUROC of 0.7 was found for the development cohort but was not reported for the validation cohort. Again, calibration was not assessed.

Our study has several strengths. First, we included large datasets and applied the current state-of-the-art of predictive modeling. Next to the model development, we performed extensive external validation, including both temporal and geographic validation. As such, we have developed a robust prediction model to identify patients who will benefit from comprehensive medication reconciliation upon ED presentation. Second, we showed how a simple model update can be performed to increase the performance of the model in a distinct setting. Third, we only included candidate predictors that are widely and consistently available within the EHR and accessible upon ED visit. As a result, the MED-REC predictor can easily be implemented in clinical practice. Finally, we chose the presence of at least 1 clinically relevant discrepancy as the outcome variable, thereby increasing the clinical relevance of our model, and allowing us to focus on patients with the highest need for medication reconciliation.

Our study also has some limitations. First, clinical relevance of discrepancies was defined by an in-house expert panel and is therefore open to discussion. Currently, there is a notable lack of consensus regarding the classification of clinically relevant discrepancies. Leading international organizations such as the WHO and accreditation bodies like the Joint Commission International have yet to establish standardized guidelines. Therefore, we emphasize the need for international consensus or standardized indicators to guide the development of future risk prediction models. While our work represents a first step in defining clinically relevant medication discrepancies, external validation by additional experts or institutions (eg, the World Academic Council of Emergency Medicine) is warranted. Second, the requirement of informed consent introduced the possibility of volunteer bias, where individuals who choose to participate may differ systematically from those who do not, possibly affecting the generalizability of our results. However, only a minority of screened patients refused informed consent (ie, 3.4%, 1.3%, and 5% in the development, temporal, and geographic validation cohort, respectively). Third, our enrollment protocol, for example, starting with the patients who have been present the longest, may have introduced selection bias, as they might present with more complex pathology, which might reduce the generalizability of our results. We chose this approach to ensure the inclusion of patients presenting during nighttime or on weekends, aiming to reduce potential bias related to the timing of ED presentation. Although we recognize that this approach may introduce other forms of bias, we think it remains limited. For instance, very complex patients can be both transferred very quickly (eg, a patient having a major cardiovascular event that is transferred immediately to the catheterization laboratory) as well as stay in the ED for an extended duration (eg, a patient with prostatitis who clinically deteriorates and is challenging to transfer). Finally, additional variables, which might further increase the performance of our model, may be missing. Variables that can be assessed in future research include the presence of specific comorbidities and multimorbidity [29], ED crowding, the day of ED presentation and whether it is a holiday or weekend, the number of prior hospital visits and revisits, and the date of the last medication history validation. We did not include these variables in this study because they were either not structurally available in our EHR, not documented, or not prevalent. The presence of multiple comorbidities would likely increase the risk of medication discrepancies due to the complexity of the patient’s medical regimen and the likelihood of polypharmacy. Nonetheless, the number of preadmission drugs and preadmission drugs per ATC code might have partially captured comorbidities in our study. High patient volumes and staff workload during periods of crowding might lead to rushed or incomplete medication reconciliation, increasing the risk of discrepancies.

### Conclusions

The probability of having at least 1 clinically relevant medication discrepancy can be calculated by the MED-REC predictor with moderate performance. The MED-REC predictor outperforms medication reconciliation at random or based on other typical selection criteria, offering a guide for the rational use of limited resources at the ED. Depending on available resources, different probability thresholds can be applied to increase either the specificity or the sensitivity of the MED-REC predictor.

## Appendix

Multimedia Appendix 1 Structured form for medication reconciliation.

Multimedia Appendix 2 Supplementary methods: collection of patient-, drug-, emergency department visit–, and medication reconciliation–related factors.

Multimedia Appendix 3 Supplementary figures: decision curve analyses for the MED-REC predictor in the temporal and geographic validation dataset.

## Abbreviations

ATC: Anatomical Therapeutic Chemical
AUROC: area under the receiver operating characteristic curve
AZ Sint-Jan Brugge-Oostende: General Hospital Sint-Jan Brugge-Oostende AV
c-index: concordance index
ED: emergency department
EHR: electronic health record
ESI: Emergency Severity Index
TRIPOD: Transparent Reporting of a Multivariable Prediction Model for Individual Prognosis or Diagnosis
UZ Leuven: University Hospitals Leuven
WHO: World Health Organization

## Data Availability

The datasets generated and analyzed during this study are available from the corresponding author on reasonable request.
