# Supplementary material for: A Prediction Model to Identify Clinically Relevant Medication Discrepancies at the Emergency Department (MED-REC Predictor): Development and Validation Study
Source: J Med Internet Res. 2024 Nov 27;26:e55185. doi: 10.2196/55185 (PMC11635314; doi:10.2196/55185)
Supplement: Multimedia Appendix 1 [file jmir_v26i1e55185_app1.docx]

**Structured Form Medication Reconciliation**

Check and/or circle:

🞎 “Are you taking any drugs?" YES – NO

🞎 “Did you recently (<2w) take any drugs?" YES – NO

🞎 “Who takes care of your drugs?” PATIENT – OTHER PERSON:

Who?________________________________

🞎 “Did you bring your drugs with you?” YES - NO:

 Ask: “is it everything?”

 Inspect drugs/packages

 Original package? YES – NO: ________________________________

🞎 “Did you bring your medication list with you?” YES - NO:

How recent is the list? ________________________________

🞎 Check medication with patient

| Drug | Dose | Route | Schedule | Time | Brought drug? |
| --- | --- | --- | --- | --- | --- |
|  |  |  |  |  |  |
|  |  |  |  |  |  |
|  |  |  |  |  |  |
|  |  |  |  |  |  |
|  |  |  |  |  |  |
|  |  |  |  |  |  |
|  |  |  |  |  |  |
|  |  |  |  |  |  |
|  |  |  |  |  |  |
|  |  |  |  |  |  |
|  |  |  |  |  |  |
|  |  |  |  |  |  |
|  |  |  |  |  |  |

🞎 Dose: KNOWN – UNKNOWN: Call Community pharmacist: Who? .....……………… OR

Call family member: Who? .....………………………. OR

Call family doctor: Who? .....……………………………..

🞎 Half a tablet – full tablet

🞎 Medication used “as needed”:

Frequency? (once per month, once per week, …)

Max. dose?

Indication?

| **EXTRA QUESTIONS** | | | |
| --- | --- | --- | --- |
| Blood thinners?  Acetylsalicylic acid, Clopidogrel, Prasugrel, Ticagrelor, Ticlopidine  Warfarin, Acenocoumarol, Phenprocoumon  Dabigatran, Rivaroxaban, Apixaban, Edoxaban |  | Recent (<2weeks) use of antibiotics? Antimycotics? |  |
| Drugs for reflux?  Calcium/magnesium carbonate,  Omeprazole, Pantoprazole, Esomeprazole |  | Sleeping pills? |  |
| Eye preparations?  Bilateral, left, right |  | Syringes?  Enoxaparin,Nadroparin?  Insulins? |  |
| Pain pills?  Paracetamol, Acetylsalicylic acid, Ibuprofen, Tramadol |  | Supplements? Vitamines? |  |
| Patches?  Patch-free period?  Full patch or cut in half? |  | Hormonal products?  Young women: pill, ring, implant, patch, intrauterine device  Man: drugs for prostate? |  |
| Aerosols? |  | Dermal preparations? |  |
| If Aerosols, steroids? |  | Magisterial preparations made by pharmacist? |  |
| Medication taken 1x/week? 1x/month? |  | *If methotrexate, folinic acid?* |  |
|  |  | *If paralyzed patient, baclofen?* |  |

🞎Repeat and summarize!

How many drugs do you take in the morning, in the afternoon, in the evening?

🞎Contacts

 pharmacist ________________________________________________

 shared pharmaceutical file: YES – NO

 medication scheme: YES – NO

 general practitioner (GP) ____________________________________________

 family member / close relative _____________________________________________
