# Supplementary material for: A Prediction Model to Identify Clinically Relevant Medication Discrepancies at the Emergency Department (MED-REC Predictor): Development and Validation Study
Source: J Med Internet Res. 2024 Nov 27;26:e55185. doi: 10.2196/55185 (PMC11635314; doi:10.2196/55185)
Supplement: Multimedia Appendix 2 [file jmir_v26i1e55185_app2.docx]

**Supplementary Methods**

*Patient-, drug-, emergency department visit-, and medication reconciliation-related factors*

A structured form was used to collect patient-, drug, emergency department visit- and medication reconciliation-related factors. Patient-related factors were sex, age, spoken language (Dutch/French/English), residence before ED admission (home/nursing home/others), transport to the ED (patient’s own transport/ambulance/emergency physician transport vehicle) and ED triage acuity scale. Drug-related factors were the number of drugs already documented by the ED physician on admission, the number of drugs classified based on the WHO’s Anatomical Therapeutic Chemical (ATC) classification system, and the number of high-risk drugs. High-risk drugs were classified according to the Institute for Safe Medication Practices’ high-alert list and the North Carolina Narrow Therapeutic Index list (i.e., drugs used in diabetes, antithrombotic agents, cardiac glycosides, procainamide, levothyroxine, antivirals for systematic use, antineoplastic agents, immunosuppressants, opioids, antiepileptics, lithium, theophylline)(1, 2) Factors related to the emergency department visit were time of ED visit (morning/afternoon/evening/night), the specialization of the physician obtaining the medication history at presentation (emergency medicine/internal medicine/psychiatry/surgery). Factors related to the medication reconciliation process were the availability of a regionally shared electronic medication record (yes/no) and electronic validation of the medication history by the ED physician (yes/no).

1. *Institute for Safe Medication Practices (ISMP).   ISMP List of High-Alert Medications in Community/Ambulatory Care Settings.*: ISMP; 2021.

2. North Carolina Board of Pharmacy. North Carolina Narrow Therapeutic Index list.
