# Supplementary material for: A Prediction Model to Identify Clinically Relevant Medication Discrepancies at the Emergency Department (MED-REC Predictor): Development and Validation Study
Source: J Med Internet Res. 2024 Nov 27;26:e55185. doi: 10.2196/55185 (PMC11635314; doi:10.2196/55185)
Supplement: Multimedia Appendix 3 [file jmir_v26i1e55185_app3.docx]

**Supplementary Figures**


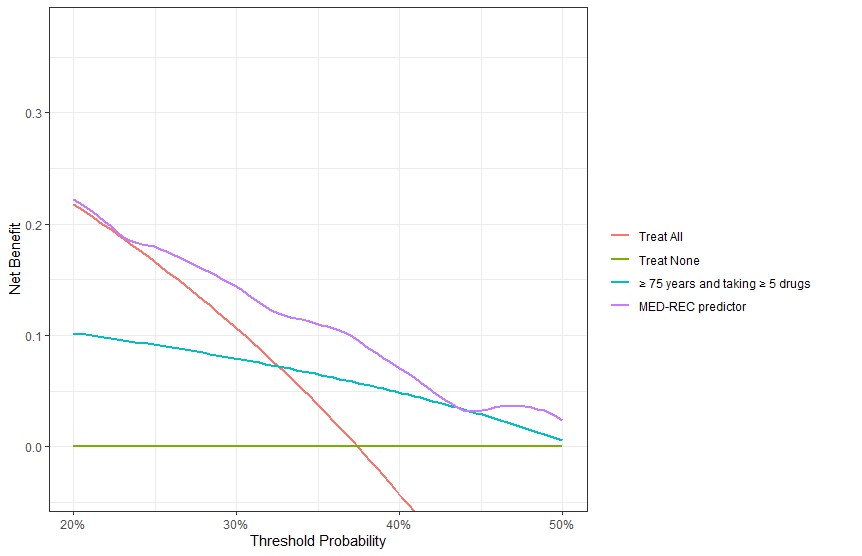


**Figure S1.** Decision curve analysis for the MED-REC predictor in the temporal validation dataset. Red line (treat all): all patients receive medication reconciliation. Green line (treat none): none of the patients receives medication reconciliation. Blue line: selection of patients who are ≥75 years and take ≥5 drugs. Purple line: MED-REC predictor. The MED-REC predictor shows net benefit over the range of clinically reasonable threshold probabilities.


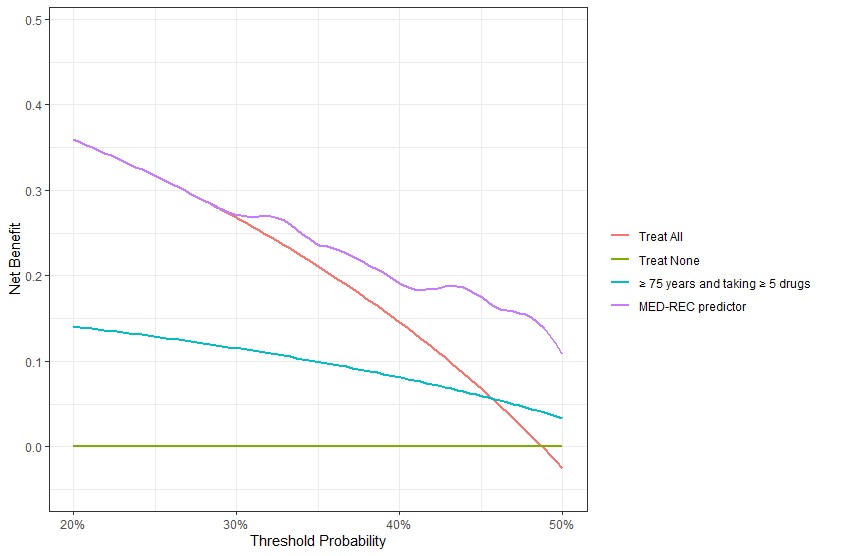


**Figure S2**. Decision curve analysis for the updated MED-REC predictor in the geographic validation dataset. Red line (treat all): all patients receive medication reconciliation. Green line (treat none): none of the patients receives medication reconciliation. Blue line: selection of patients who are ≥75 years and take ≥5 drugs. Purple line: MED-REC predictor. The updated MED-REC predictor shows net benefit starting from a probability threshold of 30%.


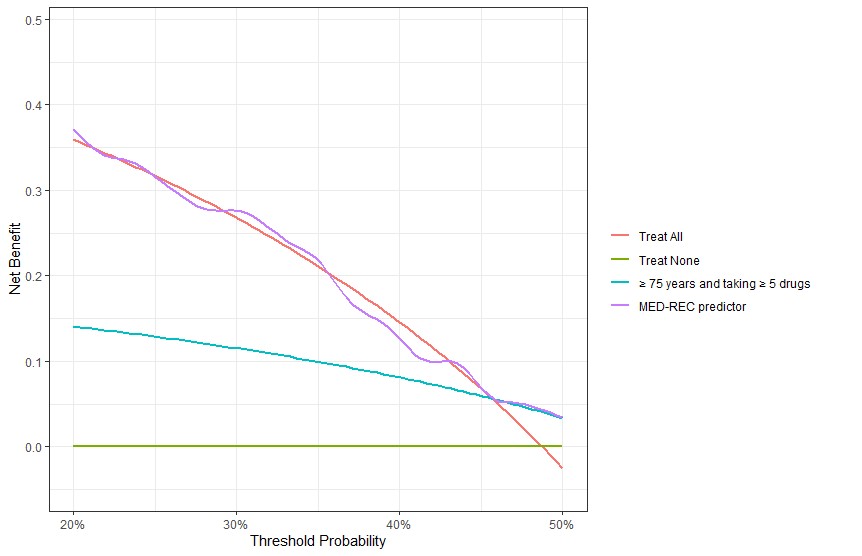


**Figure S3.** Decision curve analysis for the miscalibrated MED-REC predictor in the geographic validation dataset. Red line (treat all): all patients receive medication reconciliation. Green line (treat none): none of the patients receives medication reconciliation. Blue line: selection of patients who are ≥75 years and take ≥5 drugs. Purple line: MED-REC predictor. The miscalibrated MED-REC predictor does not show net benefit.
